# Prediction of circRNA-Disease Associations Based on the Combination of Multi-Head Graph Attention Network and Graph Convolutional Network

**DOI:** 10.3390/biom12070932

**Published:** 2022-07-02

**Authors:** Ruifen Cao, Chuan He, Pijing Wei, Yansen Su, Junfeng Xia, Chunhou Zheng

**Affiliations:** 1 Information Materials and Intelligent Sensing Laboratory of Anhui Province, School of Computer Science and Technology, Anhui University, Hefei 230601, China; hechuan9712@163.com; 2 Institutes of Physical Science and Information Technology, Anhui University, Hefei 230601, China; weipj@ahu.edu.cn (P.W.); junfeng.xia@foxmail.com (J.X.); 3 School of Artificial Intelligence, Anhui University, Hefei 230601, China; suyansen@ahu.edu.cn

**Keywords:** circular RNAs, circRNA-disease associations, graph attention network, random walk with restart algorithm, graph convolutional network

## Abstract

Circular RNAs (circRNAs) are covalently closed single-stranded RNA molecules, which have many biological functions. Previous experiments have shown that circRNAs are involved in numerous biological processes, especially regulatory functions. It has also been found that circRNAs are associated with complex diseases of human beings. Therefore, predicting the associations of circRNA with disease (called circRNA-disease associations) is useful for disease prevention, diagnosis and treatment. In this work, we propose a novel computational approach called GGCDA based on the Graph Attention Network (GAT) and Graph Convolutional Network (GCN) to predict circRNA-disease associations. Firstly, GGCDA combines circRNA sequence similarity, disease semantic similarity and corresponding Gaussian interaction profile kernel similarity, and then a random walk with restart algorithm (RWR) is used to obtain the preliminary features of circRNA and disease. Secondly, a heterogeneous graph is constructed from the known circRNA-disease association network and the calculated similarity of circRNAs and diseases. Thirdly, the multi-head Graph Attention Network (GAT) is adopted to obtain different weights of circRNA and disease features, and then GCN is employed to aggregate the features of adjacent nodes in the network and the features of the nodes themselves, so as to obtain multi-view circRNA and disease features. Finally, we combined a multi-layer fully connected neural network to predict the associations of circRNAs with diseases. In comparison with state-of-the-art methods, GGCDA can achieve AUC values of 0.9625 and 0.9485 under the results of fivefold cross-validation on two datasets, and AUC of 0.8227 on the independent test set. Case studies further demonstrate that our approach is promising for discovering potential circRNA-disease associations.

## 1. Introduction

Circular RNAs (circRNAs) produced by reverse splicing of pre-mRNAs are single-stranded, covalently closed RNA molecules, which are lacking a 5′ cap and a 3′ polyadenylated tail [1]. However, the original circRNAs were only known to be junk from splicing errors [2], and without any function. Due to the rapid development of high-throughput RNA sequencing technology, researchers have unearthed numerous circRNAs. Moreover, experimental research showed that some circRNAs are highly expressed in specific types of tissues and cells [3,4]. The expression of hundreds of circRNAs also changes during the epithelial-mesenchymal transformation of human cells [5]. These studies indicate that circRNAs are not junk from splicing errors; instead, they have irreplaceable biological functions. The explored experiments [6,7] showed that circRNAs are involved in the occurrence of various complex diseases. To be specific, circular RNAs are closely associated with cancer [8]. For example, biological exploration has shown that the proliferation and invasion of gastric cancer cells can be prevented by inhibiting the expression of circRNAs such as has_circRNA7690_15 and hsa_circ_0047905 [9]. Yin et al. [10] found that the plasma expression level of hsa_circ_0001785 was significantly different between breast cancer patients before and after surgery and healthy individuals, which proved that it can be regarded as an emerging biomarker for breast cancer diagnosis compared with traditional biomarkers. Wang et al. [11] identified a biomarker, circCNST, as a potential biomarker for osteosarcoma patients, which significantly promoted tumor genesis of osteosarcoma cells.

In recent years, through various biological experiments and studies, more and more manually curated databases have been designed for studying circRNAs and the associations between circRNAs and diseases. These databases fall into two broad categories. The first category is databases of recording circRNA annotation resources, such as circBase [12], CircNet [13] and CircFunBase [14]. The second category is databases of circRNAs associated with diseases, such as Circ2Disease [15], CircR2Disease [16] and CircRNADisease [17]. So far, the research of circRNAs and diseases is a hot topic, and a growing number of researchers are involved in this field. However, biological experiments to determine the relationship between circRNAs and diseases are time-consuming, labor-intensive and a waste of resources. Therefore, it is important to develop calculation methods to predict the correlations between circRNAs and diseases. The existing calculation methods can be divided into two categories [18]: the method based on network and the method based on machine learning. The main process of the network-based method is to build a heterogeneous network by using circRNA-disease-related information, circRNA similar network and disease similar network, and then use corresponding network-based algorithms to deduce potential associations. For example, by constructing a heterogeneous network consisting of a circRNA similarity network, a disease similarity network and a circRNA-disease association network, Lei et al. [19] proposed a path-weighted model for predicting circRNA-disease associations (PWCDA). Fan et al. [20] constructed KATZHCDA based on the KATZ method, which calculates the similarity of nodes in heterogeneous networks using known circRNA-disease associations, the similarity of circRNA expression profiles and the similarity of disease phenotype. Based on circRNA sequence similarity and disease semantic similarity, Zuo et al. [21] built a similarity network and then put forward a kind of double matrix completion method to predict the circRNA relationship with the disease. The main process of the method based on machine learning is as follows: firstly, various features of circRNAs and diseases are manually extracted; secondly, the same number of positive and negative samples are selected; finally, machine learning methods are used to make predictions. Lei et al. [22] proposed a method called RWRKNN that uses a Random Walk with Restart algorithm and a KNN model to predict potential circRNA-disease associations. Lei et al. [23] proposed a GBDTCDA model that uses gradient enhanced decision tree and multi-biological data fusion to predict the associations between circRNA and disease. Wang et al. [24] established the GCNCDA model that uses Graph Convolutional Network fast learning to effectively extract high-quality features and further used the Forest by Penalizing Attributes algorithm to obtain the final prediction.

In this paper, we propose a novel approach based on the Multi-Head Graph Attention Network and Graph Convolutional Network to predict circRNA and disease associations, called GGCDA. Our model offers the following contributions:

1. GGCDA constructs a novel deep learning model based on multi-head GAT and GCN. Specifically, the multi-head Graph Attention Network is used to obtain different weights of circRNA and disease features, and GCN is used to aggregate the features of adjacent nodes in the network and the features of the nodes themselves. So, it can learn not only the node feature information, but also the information contained in the network structure.
2. Multiple similarities of circRNAs and diseases are integrated as the original features of nodes in the GGCDA, including circRNA sequence similarity, disease semantic similarity and corresponding Gaussian interaction profile kernel similarity.
3. RWR is used to capture the multi-faceted relationship between two nodes, capturing the overall structural information of the graph.
4. An independent dataset that contains more data is used to verify the generalization of GGCDA, besides common comparisons and case studies.

Comparisons with several state-of-the-art methods and case studies were applied to verify the performance of the proposed method; the experimental results demonstrate the capability of GGCDA for predicting circRNA-disease associations. The source code and run environment of our model are available at https://github.com/hhhhcccc22/GGCDA (accessed on 23 May 2022).

## 2. Materials and Methods

### 2.1. Known Human circRNA-Disease Associations

To measure model performance, we used CircR2Disease, an experimentally validated circRNA-disease association database. The CircR2Disease database includes 739 manually collected circRNA-disease associations from published literature, covering 661 circRNAs and 100 diseases, which can be downloaded from http://bioinfo.snnu.edu.cn/CircR2Disease/ (accessed on 23 May 2022). Based on the original CircR2Disease database, we removed duplicate associations, non-human associations and some circRNA-disease associations of circRNAs with no sequence information in the circBase database or diseases with no disease ontology identity information in the Disease Ontology (DO) database [25]. Consequently, from the CircR2Disease dataset we used, there are a total of 651 associations, including 590 circRNAs and 88 diseases. 

To validate the stability of the model, we constructed a larger dataset called DATA containing more data information from three databases: CircR2Disease, Circ2Disease and CircRNADisease. After de-duplication and the same operation as above, we integrated the data from these three databases, and finally, the DATA dataset containing 944 associations of 809 circRNAs and 119 diseases was obtained.

To investigate the generalization of the model, we tested the models on an independent dataset that was used in the reference [26]. We collected circRNA-disease associations from the circRNA-disease pair information in the database MNDR version 3.1 [27] and removed the circRNA-disease pairs that are not human. Finally, a total of 2175 kinds of circRNAs and 154 kinds of diseases were obtained with 2758 circRNA-disease pairs. All dataset information can be seen in Table 1.

In order to facilitate the expression of the calculation formula, we used $A\in {\mathbb{R}}^{nc\times nd}$ to represent the association matrix between circRNAs and diseases, where nc and nd are the numbers of circRNAs and diseases, respectively. When a circRNA $c_{i}$ is correlated with a disease $d_{j}$ in the dataset, we set the association matrix $A_{i,j}$ at the corresponding position to have the value 1; otherwise, the value is 0. 

### 2.2. CircRNA Sequence Similarity

To obtain the sequence similarity of circRNAs, we downloaded all the human circRNAs in the circBase database, which contains 140,790 pieces of circRNA sequence information. Then, we extracted the circRNAs sequence information corresponding to the circRNA-disease associations. The similarity between any two circRNAs sequence is calculated based on Levenshtein distance [28]. Levenshtein distance refers to the degree of difference between two character strings, such as String1 and String2, measured by the minimum operand to convert String1 to String2 by deleting, adding, replacing, etc. A circRNA can also be regarded as a string consisting of A, G, C and T. Therefore, Levenshtein distance can be used to calculate the sequence similarity in the field of biological information. The smaller the value between two circRNAs expressed by the Levenshtein distance, the greater the similarity. The similarity of circRNA $c_{i}$ and circRNA $c_{j}$ is calculated as follows:

(1) $$SC_{seq}(c_{i},c_{j})=1-\frac{LevDis(c_{i},c_{j})}{length(c_{i})+length(c_{j})}$$

where $LevDis(c_{i},c_{j})$ represents the operand to convert circRNA $c_{i}$ to circRNA $c_{j}$, and $length(c_{i})$ represents the sequence length of circRNA $c_{i}$.

### 2.3. Disease Semantic Similarity

To calculate the semantic similarity of diseases, we downloaded disease terms including DOID, name and associations with parents from the DO database. Given a disease d, a directed acyclic graph (DAG) can be formed, which can be expressed as $DAG_{d}(F_{d},E_{d})$, where $F_{d}$ represents the disease node and $E_{d}$ represents the relationship between the various disease nodes. Based on Wang’s summary of the ontology of disease [29], we used the DOSE function in the R package to calculate the similarity between disease pairs, represented by $SD_{ds}\in {\mathbb{R}}^{nd\times nd}$; its calculation formula is as follows:

(2) $$SD_{ds}(d_{i},d_{j})=\frac{\sum_{f\in F_{d_{i}}\cap F_{d_{j}}}\left(D_{d_{i}}(t)+D_{d_{j}}(t)\right)}{\sum_{f\in F_{d_{i}}}D_{d_{i}}(t)+\sum_{f\in F_{d_{j}}}D_{d_{j}}(t)}$$

where $F_{d_{i}}$ means disease $d_{i}$ and its ancestors in DAG. $D_{d_{i}}$ represents the contribution value of all nodes in $F_{d_{i}}$ to disease $d_{i}$, which can be explained by the following formula:

(3) $$\left\{ \begin{array}{l} D_{d}(t)=1,if\;t=d\\ D_{d}(t)=\max\left\{\psi \cdot D_{d}(t^{,})|t^{,}\in children\;of\;t\right\},if\;t\neq d \end{array}\right.$$

where ψ represents the disease semantic contribution score, which is set to 0.5 according to Wang’s method [29].

### 2.4. Gaussian Interaction Profile Kernel Similarity for circRNA and Disease

Due to the sparse sequence similarity of circRNAs and the semantic similarity of diseases, we introduced the Gaussian interaction profile kernel [30] (GIP) similarity that is constructed to measure circRNA similarity and disease similarity based on the known circRNA-disease association matrix A defined in Section 2.1. In A, the ith row K(i) represents the association of the circRNA with all diseases, and it is a binary vector. The jth column K(j) means the association of the jth disease with all circRNAs. Based on the assumption that similar diseases are more likely to be associated with similar circRNAs and vice versa, we calculate the GIP similarity as follows:

(4) $$SC_{GIP}(c_{i},c_{j})=\exp(-\rho_{c}{\left\|K(c_{i}-K(c_{j})\right\|}^{2})$$

(5) $$SD_{GIP}(d_{i},d_{j})=\exp(-\rho_{d}{\left\|K(d_{i})-K(d_{j})\right\|}^{2})$$

where $\rho_{c}$ and $\rho_{d}$ are utilized to control the kernel bandwidth, and the calculation formulas can be expressed as follows:

(6) $$\rho_{c}=1/\left(\frac{1}{n_{c}}\sum_{i=1}^{n_{c}}{\left\|K(c_{i})\right\|}^{2}\right)$$

(7) $$\rho_{d}=1/\left(\frac{1}{n_{d}}\sum_{j=1}^{n_{d}}{\left\|K(d_{j})\right\|}^{2}\right)$$

where $n_{c}$ represents the number of rows indicating the number of circRNAs, and $n_{d}$ represents the number of columns indicating the number of diseases.

### 2.5. Integration of Different Similarities

In order to solve the problem that single similarity of circRNAs and diseases is too sparse and causes the instability of the prediction results, we combined the GIP similarity of circRNAs with sequence similarity, and the GIP similarity of diseases with semantic similarity, respectively. The combination formula is as follows:

(8) $$SC_{Fus}(c_{i},c_{j})=\left\{ \begin{array}{l} SC_{seq}(c_{i},c_{j}),if\;SC_{seq}(c_{i},c_{j})\neq 0\\ SC_{GIP}(c_{i},c_{j}),else \end{array}\right.$$

(9) $$SD_{Fus}(d_{i},d_{j})=\left\{ \begin{array}{l} SD_{ds}(d_{i},d_{j}),if\;SD_{ds}(d_{i},d_{j})\neq 0\\ SD_{GIP}(d_{i},d_{j}),else \end{array}\right.$$

where $SC_{seq}$ and $SC_{GIP}$ represent circRNA sequence similarity and GIP similarity, respectively, and $SD_{ds}$ and $SD_{GIP}$ represent disease semantic similarity and GIP similarity, respectively.

### 2.6. Feature Initialization of circRNAs and Diseases with RWR

As mentioned above, $SC_{Fus}$ and $SD_{Fus}$, as the similarity profile of circRNA and disease, can be used as the feature vector of circRNA and disease, respectively. However, due to the limitation of the similarity calculation method of circRNA and disease, $SC_{Fus}$ and $SD_{Fus}$ alone may not be sufficient and may lead to missing the structure of the network. Therefore, we apply RWR to obtain topological context vectors of circRNAs and diseases from $SC_{Fus}$ and $SD_{Fus}$. RWR aims to capture the overall structure of the graph information, which starts from a node in the graph. Each step is faced with two choices: randomly select adjacent nodes or return to the start node; we apply the RWR algorithm to process $SC_{Fus}$ and $SD_{Fus}$, respectively, and its formula can be expressed as:

(10) $$P_{i}^{k+1}=(1-\theta )P_{i}^{k}\hat{S}+\theta P_{i}^{0}$$

where $P_{i}^{k}$ represents the ith row vector after k update operations. θ represents the restart probability; based on previous research, we set it to 0.9 [31]. $\hat{S}$ represents the one-step probability transition matrix after normalizing the similarity matrix $SC_{Fus}$ (or $SD_{Fus}$) by column, $P_{i}^{0}$ is a binary vector and $P_{i,i}^{0}=1$, else, 0. Finally, SC and SD are used to represent the new feature after the RWR algorithm.

### 2.7. CircRNA-Disease Heterogeneous Network Construction

**Figure 1:**
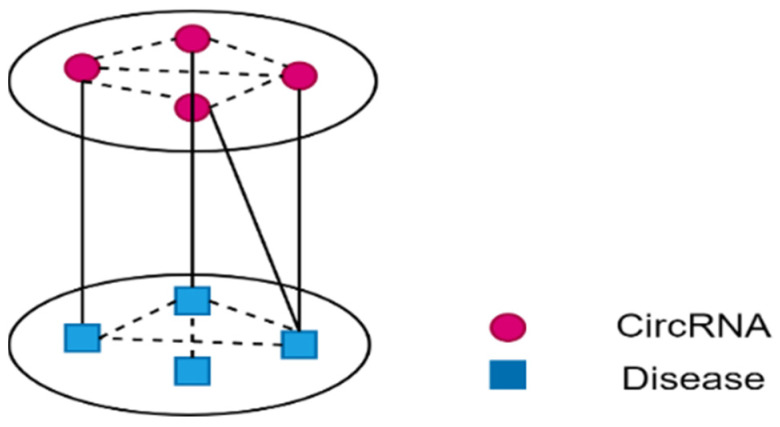
Illustration of the heterogeneous network.

Based on the above data processing, we constructed a heterogeneous network (called $G_{Het}$) consisting of circRNA-disease association network, circRNA feature vector and disease feature vector. The edges between circRNAs (or diseases) represent their similarity, and the edges between circRNAs and diseases represent associations. Meanwhile, we introduced a penalty factor μ to control the contribution of similarity to the model. The construction expression of the heterogeneous network $G_{Het}\in {\mathbb{R}}^{\left(nc+nd)\times (nc+nd\right)}$ can be shown as Equation (11), and its representation is shown in Figure 1.

(11) $$G_{Het}=\left[\begin{array}{ll} \mu \cdot SD & A\\ A^{Τ} & \mu \cdot SC \end{array}\right]$$

### 2.8. The Framework of the Proposed GGCDA

**Figure 2:**
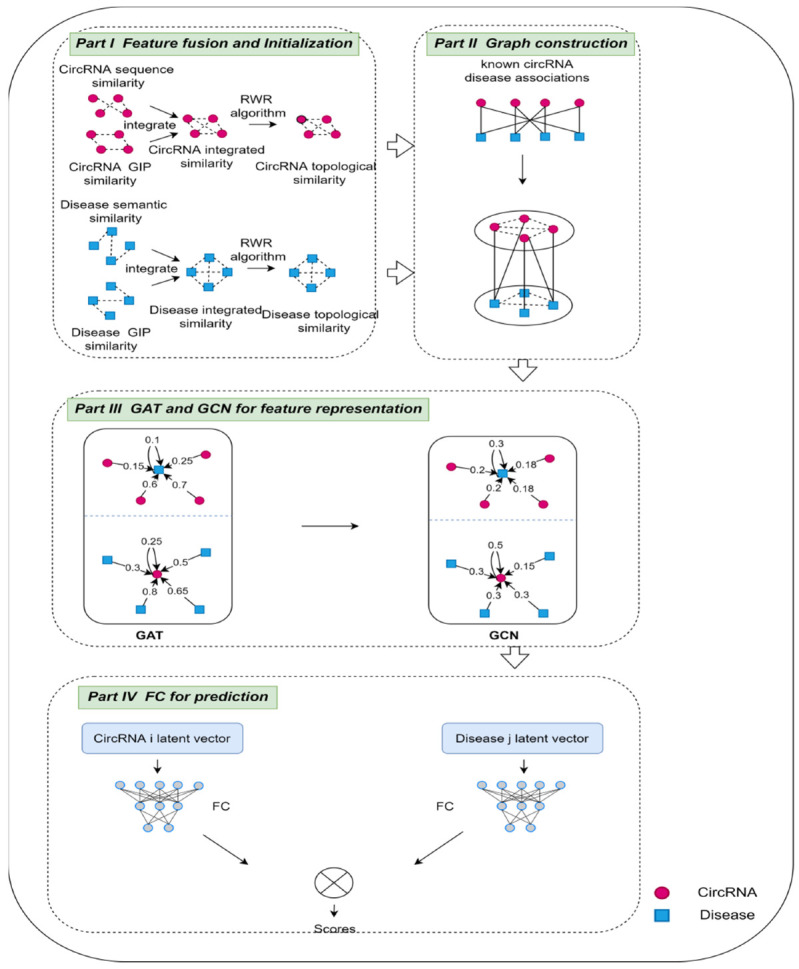
The framework of GGCDA, composed of four parts: (I) feature fusion and initialization; (II) circRNA-disease heterogeneous network construction; (III) features representation based on the combination of multi-head GAT and GCN; (IV) fully connected layer for prediction.

To obtain potential circRNA-disease associations, relying on the above heterogeneous network, we constructed a hybrid model based on multi-head GAT and GCN, as shown in Figure 2. The framework of GGCDA contains four parts. Firstly, multiple similarities are integrated, and RWR is used to introduce the topological context of each circRNA and disease node into its initial feature representation. Secondly, based on the known association matrix between circRNAs and diseases, and the initial feature of the circRNA and disease node, a heterogeneous network is constructed. Thirdly, a combination of the multi-head GAT and GCN is proposed to extract different weight features of circRNA and disease features using the multi-head GAT, and then GCN is applied to aggregate the features of adjacent nodes and the features of the nodes themselves to obtain multi-view circRNA and disease features. Finally, we implemented a fully connected layer network to identify potential associations. As the first two parts of the framework are given above, we present the key issues in the last two parts in the following.

#### 2.8.1. Features Representation Based on the Combination of Multi-Head GAT and GCN

A graph neural network (GNN) is aimed at learning high-level feature representation vectors from a graph structure [32]. In applications in the field of bioinformatics, GNN methods are widely used, such as drug-target associations (DTA) [33] and ncRNA-protein interactions (NPI) [34]. In this study, we use the GAT [35] and GCN [36], which can learn the embedding of nodes more effectively.

Based on the heterogeneous graph $G_{Het}$, we define adjacency matrix M and feature matrix X as follows:

(12) $$M=\left[\begin{array}{ll} 0 & A\\ A^{Τ} & 0 \end{array}\right]$$

(13) $$X=\left[\begin{array}{ll} \mu \cdot SD & 0\\ 0 & \mu \cdot SC \end{array}\right]$$

GAT introduced the self-attention mechanism, which represents the weight of adjacent nodes by the attention coefficient, so as to learn the hidden representation of nodes on the graph. To be specific, to learn the importance of the first-order neighbor nodes to a particular node, GAT calculates the attention coefficient for each node through a forward linear transformation, where the attention coefficient $e_{ij}$ between circRNA $c_{i}$ and disease $d_{j}$ can be expressed as the following formula:

(14) $$e_{ij}(c_{i},d_{j})=f(Wb_{i},Wb_{j})$$

where f represents a single-layer neural network, W is a weight matrix, b is the feature representation of the current node and $b_{0}$ is the initial feature matrix X. Then, the normalized attention coefficient $\alpha_{i,j}$ is obtained by computing the softmax function as follows:

(15) $$\alpha_{\mathrm{ij}}=\frac{\exp(e_{ij})}{\sum_{l\in N_{i}}\exp(e_{il})}$$

where $N_{i}$ represents the first-order neighbors of the node i.

The output result of the final node was shown as follows:

(16) BNi=Relu(∑t∈NiαijWbt)

where $N_{i}$ represents the neighbor nodes of the current node, and α represents the normalized attention coefficient. In order to improve its robustness, multi-head attention GAT was used in the proposed model; the results are expressed through N-head multi-head GAT layer as follows:

(17) BNi=Relu(1N∑i=1N∑t∈NiαitiWibt)

However, different from GAT, GCN can learn the representation of nodes on the graph level. Its basic idea is to conduct convolution operations on the graph to obtain node embedding. After obtaining the attention features through the GAT, GCN is used to fully aggregate node information from its neighbors and its own node information on circRNA and disease feature, so as to update node features and generate embedding. The specific embedding representation is shown as follows:

(18) Hl+1=Relu(D˜−12M˜D˜−12HlWl)M˜=M+ID˜ii=∑jM˜ij

where I represents the identity matrix, M represents the circRNA-disease adjacency matrix, W represents the weight matrix, $H^{l}$ represents the feature of the lth layer of circRNA and disease and $H^{0}$ is the initial feature matrix X.

#### 2.8.2. Fully Connected Layer for Prediction

The above GAT layer and GCN layer can be performed multiple times. Through the hybrid GNN of GAT and GCN, we obtain the potential feature representation of diseases and circRNA. To predict potential circRNA-disease associations, we use fully connected neural networks as classifiers for prediction, as they are widely used in classification. In the previous l−1 layer, we calculate the circRNAs (or diseases) results using the following formula:

(19) F(x+1)=Relu(WxFx+bx)

where x should be less than or equal to l−2, and W and b represent weight parameters and bias, respectively. The output result of layer l can be expressed as: $S(c,d)=F^{l}=sigmoid(W^{l-1}F^{l}+b^{l})$, where $sigmoid=\frac{1}{1\;+\;e^{-x}}$; it maps the result variable between 0 and 1.

According to the previous study [37], we set l=3 in our model.

#### 2.8.3. Loss Function

In the process of model training, we optimize the loss function by cross entropy loss and L2 regularization; here, we randomly select negative sample data with an equal number of positive samples from the association matrix, which may be potential candidates but are labeled as 0, where the loss function defined as follows:

(20) $$Loss=-\frac{1}{N}(\sum_{i,j}y_{ij}\log S(c_{i},d_{j})+(1-y_{ij})\log(1-S(c_{i},d_{j})))+\frac{\lambda }{2}||\Theta |{|}^{2}$$

where N represents the number of training sets, y is the actual label, $S(c_{i},d_{j})$ is the prediction result, λ is the control factor, Θ represents all the parameters in the model, and W is the weight matrix that can be learned in the forward neural network.

## 3. Results

### 3.1. Evaluation Criteria

In this study, fivefold cross-validation (FFCV) was used to evaluate the performance of the GGCDA model. Specifically, we randomly divided all samples into five subsets. In each fold, four subsets are used as training sets and one subset is used as the test set. Here, the known circRNA-disease pairs are regarded as positive samples, whereas the randomly selected unknown circRNA-disease pairs with the same number of positive samples are negative samples.

We adopted evaluation indexes that are frequently used to evaluate the performance of the machine learning model, including Accuracy (ACC), Precision (PRE), Recall (REC) and F1-Score. The larger the above evaluation index value is, the more outstanding the performance of the model is. In order to further explore the model effect, we describe the receiver operating characteristic curve (ROC) and precision-recall curve (PR). From these, the area under the receiver operating characteristic curve (AUC) [38] and the area under the precision-recall curve can be calculated (AUPR) [39] to fully reflect the performance of the model. 

### 3.2. Prediction Performance

**Figure 3:**
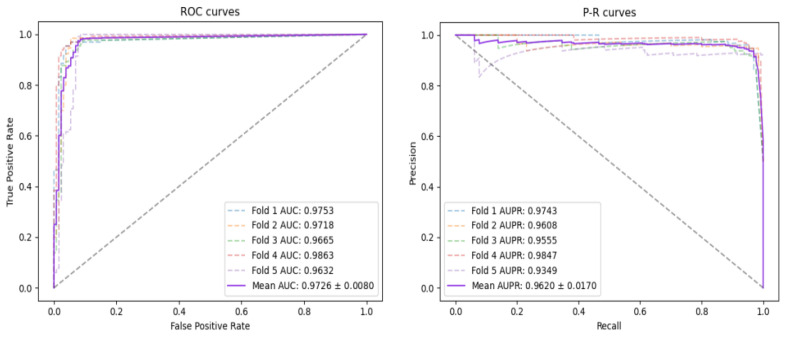
AUC and AUPR curves of FFCV obtained by GGCDA on CircR2Disease.

Since the random allocation of negative samples may lead to slight errors in the experimental results, we repeated the experiment 10 times. From the experimental results listed in Table 2, on the CircR2Disease database, the AUC and AUPR of FFCV using GGCDA can reach 0.9726 and 0.9620, respectively, and ACC, PRE, REC and F1-Score results are 0.9063, 0.8511, 0.9861 and 0.9134, respectively. Furthermore, we drew the ROC curve and PR curve of the model under the CircR2Disease database, as shown in Figure 3. From Table 2, we also can see the average results of the 10 FFCVs: the values of AUC, AUPR, ACC, PRE, REC and F1-Score obtained by GGCDA are 0.9625, 0.9422, 0.9172, 0.8700, 0.9822 and 0.9224, respectively.

### 3.3. Parameter Analysis

In this part, we discuss the impact of model parameters on the performance of GGCDA based on the CircR2Disease database, including the number of attention heads, the number of GAT layers, the number of GCN layers, embedding numbers and the penalty factors.

**Figure 4:**
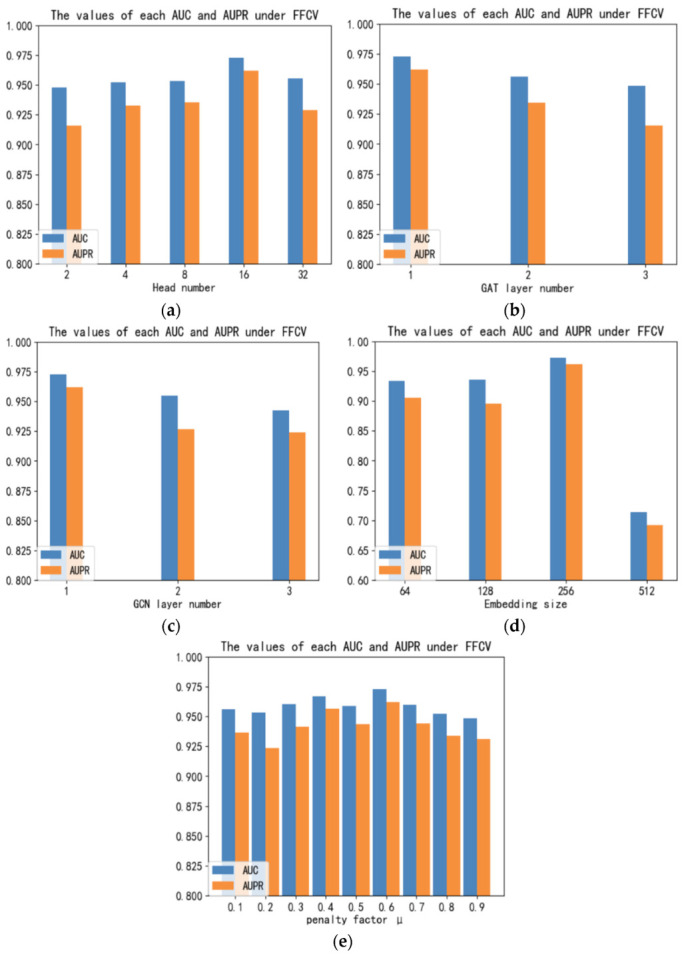
Impact of different parameters on model performance: (**a**) Comparison of AUC and AUPR values for different attention heads. (**b**) Comparison of AUC and AUPR values at different GAT layers. (**c**) Comparison of AUC and AUPR values at different GCN layers. (**d**) Comparison of AUC and AUPR values at different embedding sizes. (**e**) Comparison of AUC and AUPR values on different penalty factors.

Specifically, the attention model using multiple heads in the GAT layer will be more robust. As shown in Figure 4a, we set the number of heads as (2, 4, 8, 16, 32). It can be seen that GGCDA can achieve better performance with the increase in the number of attention heads, but the performance will decline when the number exceeds 16. So, we set the number of heads of GAT to 16. In addition, the number of layers of GAT and GCN will also have an impact on the model. We set the number of layers for these two components, and the experimental results as shown in Figure 4b,c. It can be seen that GAT and GCN both perform best when the number of layers is 1. When the number of layers increases, GGCDA cannot learn much information. Finally, we explore the impact of embedding on the model effect. Different from previous studies, we set the embedding number as (64, 128, 256, 512). As shown in Figure 4d, when the embedding number is 256, GGCDA can achieve the best performance. We also set the penalty factors from 0.1 to 0.9, and the results from Figure 4e show that when μ is equal to 0.6, it contributes the most to the performance of the model.

### 3.4. Model Ablation Study

Since our model is composed of several sections, in this part, ablation studies were conducted based on the CircR2Disease database. In order to judge the influence of each component on the overall performance of the model, FFCV was performed, and ablation studies were mainly conducted on the following variants.

1. GGCDA without RWR: only circRNA similarity matrix and disease similarity matrix are used instead of features calculated after RWR.
2. GGCDA without GAT: only the convolution features extracted by GCN are used.
3. GGCDA without GCN: only features with attention mechanisms extracted by GAT are used.
4. GGCDA without FC: use the inner product instead of FC to predict.

**Figure 5:**
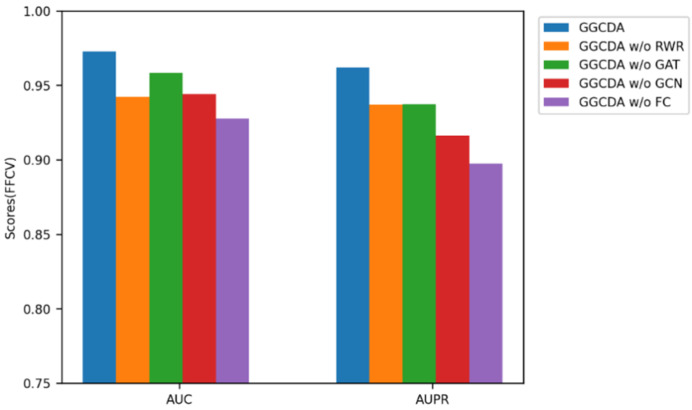
Performance compared with different component.

Figure 5 shows the performance comparison between each model variant and GGCDA. Compared with only GAT or GCN, after combining GAT and GCN, GGCDA can achieve better performance. At the same time, for GGCDA without FC, the performance of the model is also relatively low, indicating the important role of distillation. Moreover, the performance degradation after GGCDA without RWR also shows that introducing RWR as part of the model is an important choice.

### 3.5. Comparisons with Existing Methods

So far, a growing number of researchers have designed numerous models to predict circRNA-disease associations. To demonstrate the superiority of GGCDA, we calculated the AUC values against recently published models on the CircR2Disease dataset, including DMCCDA [21], NCPCDA [40], GCNCDA [24], RWRKNN [22] and GATCDA [39].

In this section, we also used the value of AUC as a comparison indicator. It can be seen from Table 3 that on the dataset CircR2Disease, GGCDA had the best AUC value of 0.9625; other models’ AUC values were 0.9598, 0.9201, 0.9090, 0.9333 and 0.9011. We found that the results of DMMCDA are similar to ours, but the results are still slightly behind ours. So, it can be inferred that GGCDA based on the combined GAT and GCN achieves optimal performance and is a promising approach.

### 3.6. Performance on a Larger Dataset

In order to further verify the robustness of the model and explore the performance on a large dataset, we performed FFCV on the DATA dataset.

**Figure 6:**
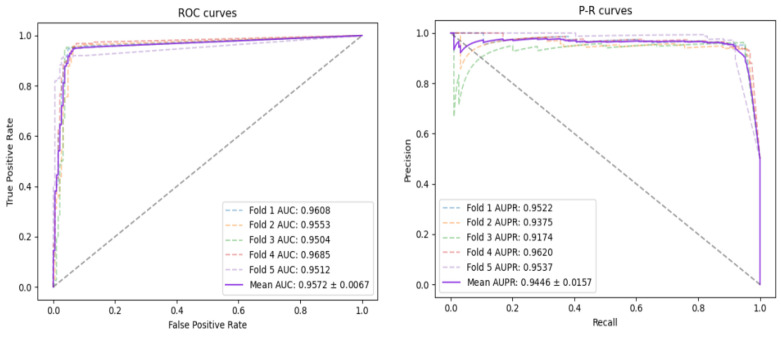
AUC and AUPR curves of FFCV obtained by GGCDA on DATA.

In this experiment, the composition of the model we adopted is the same as the previous one, and there are only differences in the data. The detailed results can be seen in Table 4. The ROC curve and PR curve of the model is shown in Figure 6. It can be seen from the experimental results that the change in the amount of data does not affect the performance of the model to a certain extent. AUC, AUPR, ACC, PRE, REC and F1-Score reach 0.9485, 0.9266, 0.9116, 0.8827, 0.9505 and 0.9150, respectively. The analysis of the experimental results shows that although the amount of data may affect the performance of the model, GGCDA still has certain robustness.

### 3.7. Results on the Independent Test Set

To investigate the generalization of the model, we tested the model on an independent test set, the dataset MNDR, which is described in detail in Section 2.1 and Table 1. We use the GGCDA model to predict the samples of the dataset MNDR, and the results are shown in Table 5. It can be seen from the results that although the performance on the independent test set is lower than the performance on the fivefold cross-validation on the other two datasets, there is not a big gap; that is, the model has a certain generalization in predicting potentially associated circRNA-disease pair ability.

### 3.8. Case Study

To further validate the superiority of GGCDA, case studies on multiple diseases were implemented. Specifically, we set all circRNA-disease samples to be validated as unknown and then used GGCDA to generate all association scores for three common diseases: hepatocellular carcinoma, breast cancer and colorectal cancer. Then, we sorted them in descending order according to the scoring of the model, and finally screened out the top 10 circRNAs corresponding to these diseases.

The results of case studies on hepatocellular carcinoma [41], breast cancer [42] and colorectal cancer [43] after implementing GGCDA are shown in Table 6Table 7 and Table 8. It can be seen that among the results, 8, 8 and 9 out of 10 circRNAs for hepatocellular carcinoma, breast cancer and colorectal cancer were successfully predicted and could be retrieved in the literature, respectively. The results of these experiments illustrate GGCDA’s effective performance in exploring unknown circRNA-disease associations.

## 4. Conclusions

Identifying associations between circRNAs and diseases has important implications for disease diagnosis, treatment and identification of biomarkers. In this work, we propose an effective method based on multi-head GAT and GCN for predicting potential circRNA-disease association, and use three datasets to evaluate the performance of GGCDA. Moreover, we conducted case studies on hepatocellular carcinoma, breast cancer and colorectal cancer. The experimental results demonstrate that our method has suitable performance and can effectively detect more circRNA-disease associations.

The superior performance of the GGCDA model can be attributed to several factors. Firstly, GGCDA makes full use of circRNA sequence information and integrates various information to construct a heterogeneous network. Secondly, the graph-based neural network method is used to learn the topology structure and internal information in the network of circRNAs and disease, GAT learns features on bipartite networks and GCN is embedded on weighted similarity networks, so as to obtain the representation of depth features of nodes. Moreover, the random walk with restart algorithm is used to capture the overall structure of the graph information to enhance the feature representation of the similarity network.

However, there are some limitations of our model. Firstly, GGCDA only uses circRNA sequence similarity, disease semantic similarity and Gaussian kernel similarity. In the future, we will combine more biomarkers, such as miRNA and protein, to construct more complex heterogeneous networks. With the development of the graph neural network, more and more advanced models have been proposed to learn the low-dimensional representation of graph structure, such as heterogeneous graph attention network, heterogeneous graph transformer and so on. In future research, we will continue to explore the positive role of these graph-embedding methods to identify circRNA-disease associations.

## Figures and Tables

**Table 1:** The used datasets.

Datasets	circRNA Numbers	Disease Numbers	Associations
CircR2Disease	590	88	651
DATA	809	119	944
MNDR	2175	154	2785

**Table 2:** Results of FFCV on CircR2Disease achieved by GGCDA.

Fold	AUC	AUPR	ACC	PRE	REC	F1-Score
1	0.9753	0.9743	0.8969	0.8421	0.9771	0.9046
2	0.9718	0.9608	0.9346	0.8951	0.9846	0.9377
3	0.9665	0.9555	0.9077	0.8581	0.9769	0.0136
4	0.9863	0.9847	0.8885	0.8217	0.9923	0.8989
5	0.9631	0.9349	0.9038	0.8387	1.0000	0.9123
Average	0.9726	0.9620	0.9063	0.8511	0.9861	0.9134
Average (10)	0.9625	0.9422	0.9172	0.8700	0.9822	0.9224

**Table 3:** The FFCV AUC values achieved by the various models.

Methods	DMMCDA	NCPCDA	GCNCDA	RWRKNN	GATCDA	GGCDA
AUC	0.9598	0.9201	0.9090	0.9333	0.9011	0.9625

**Table 4:** Results of FFCV on DATA achieved by GGCDA.

Fold	AUC	AUPR	ACC	PRE	REC	F1-Score
1	0.9608	0.9522	0.9312	0.9095	0.9577	0.9330
2	0.9553	0.9375	0.9339	0.9100	0. 9630	0.9357
3	0.9503	0.9174	0.9339	0.9184	0.9523	0.9351
4	0.9685	0.9620	0.9206	0.8804	0.9735	0.9246
5	0.9511	0.9537	0.9069	0.8964	0.9202	0.9081
Average	0.9572	0.9446	0.9253	0.9029	0.9534	0.9273
Average (10)	0.9485	0.9266	0.9116	0.8827	0.9505	0.9150

**Table 5:** Results of the GGCDA on the independent test set.

AUC	AUPR	ACC	PRE	REC	F1-Score
0.8227	0.7836	0.7832	0.7651	0.8173	0.7903

**Table 6:** The top 10 hepatocellular carcinoma-related candidate circRNAs.

Disease	circRNA	PMID
Hepatocellular Carcinoma	hsa_circ_0000284	29415990
	hsa_circ_0001141	28636993
	hsa_circ_0001946	28892615
	hsa_circ_0001649	26600397
	hsa_circRNA_102049	28710406
	hsa_circ_0001445	29378234
	hsa_circ_0001821	unconfirmed
	hsa_circ_0067934	29458020
	hsa_circ_0023404	unconfirmed
	hsa_circRNA_103387	28710406

**Table 7:** The top 10 breast cancer-related candidate circRNAs.

Disease	circRNA	PMID
Breast Cancer	hsa_circ_0000284	27050392
	hsa_circ_0001141	unconfirmed
	hsa_circ_0001946	28049499
	hsa_circ_0007534	29593432
	hsa_circ_0001821	27928058
	hsa_circ_0001313	28249903
	circ-Foxo3	27886165
	hsa_circ_0014717	unconfirmed
	hsa_circ_0002113	28803498
	hsa_circ_0004771	28484086

**Table 8:** The top 10 colorectal cancer-related candidate circRNAs.

Disease	circRNA	PMID
Colorectal Cancer	hsa_circ_000753	29364478
	hsa_circ_000114	26110611
	hsa_circ_000131	28249903
	hsa_circ_000182	30591054
	hsa_circ_000194	28174233
	hsa_circ_000028	27050392
	hsa_circ_000164	29421663
	hsa_circ_0067934	unconfirmed
	hsa_circ_001471	29571246
	hsa_circ_000050	28656150

## Data Availability

The circRNA-disease association database CircR2Disease is available at http://bioinfo.snnu.edu.cn/circR2Disease/ (accessed on 23 May 2022), and the Circ2Disease database is available at http://bioinformatics.zju.edu.cn/Circ2Disease/index.html (accessed on 23 May 2022). The CircRNADisease database is available at http://cgga.org.cn:9091/circRNADisease/ (accessed on 23 May 2022), and the MNDR database is available at http://www.rnadisease.org/ (accessed on 23 May 2022). The source code and data of our model are available at https://github.com/hhhhcccc22/GGCDA (accessed on 23 May 2022).
